# Multi-omics analysis reveals the host–microbe interactions in aged rhesus macaques

**DOI:** 10.3389/fmicb.2022.993879

**Published:** 2022-09-27

**Authors:** Jue Xu, Yue Lan, Xinqi Wang, Ke Shang, Xu Liu, Jiao Wang, Jing Li, Bisong Yue, Meiying Shao, Zhenxin Fan

**Affiliations:** ^1^West China School of Public Health and West China Fourth Hospital, Chengdu, Sichuan, China; ^2^Key Laboratory of Bioresources and Ecoenvironment (Ministry of Education), College of Life Sciences, Sichuan University, Chengdu, China; ^3^Sichuan Key Laboratory of Conservation Biology on Endangered Wildlife, College of Life Sciences, Sichuan University, Chengdu, China

**Keywords:** non-human primates, multi-omics, oral and gut microbiome, blood transcriptome and metabolome, aging

## Abstract

Aging is a complex multifactorial process that greatly affects animal health. Multi-omics analysis is widely applied in evolutionary biology and biomedical research. However, whether multi-omics can provide sufficient information to reveal comprehensive changes in aged non-human primates remains unclear. Here, we explored changes in host–microbe interactions with aging in Chinese rhesus macaques (*Macaca mulatta lasiota*, CRs) using multi-omics analysis. Results showed marked changes in the oral and gut microbiomes between young and aged CRs, including significantly reduced probiotic abundance and increased pathogenic bacterial abundance in aged CRs. Notably, the abundance of *Lactobacillus*, which can metabolize tryptophan to produce aryl hydrocarbon receptor (AhR) ligands, was decreased in aged CRs. Consistently, metabolomics detected a decrease in the plasma levels of AhR ligands. In addition, free fatty acid, acyl carnitine, heparin, 2-(4-hydroxyphenyl) propionic acid, and docosahexaenoic acid ethyl ester levels were increased in aged CRs, which may contribute to abnormal fatty acid metabolism and cardiovascular disease. Transcriptome analysis identified changes in the expression of genes associated with tryptophan metabolism and inflammation. In conclusion, many potential links among different omics were found, suggesting that aged CRs face multiple metabolic problems, immunological disorders, and oral and gut diseases. We determined that tryptophan metabolism is critical for the physiological health of aged CRs. Our findings demonstrate the value of multi-omics analyses in revealing host–microbe interactions in non-human primates and suggest that similar approaches could be applied in evolutionary and ecological research of other species.

## Introduction

Aging is a complex multifactorial process involving many biological pathways and molecules (Ahadi et al., 2020; Bunning et al., 2020). Aging is also a major risk factor for many chronic diseases (Lehallier et al., 2019). Therefore, a deeper understanding of aging could provide new insights into the mechanisms of age-related diseases to promote health and longevity (Alpert et al., 2019; Lehallier et al., 2019). As such, increasing studies have explored the processes and mechanisms of aging at the genomic (Kenyon, 2010; Hou et al., 2020), transcriptomic (Peters et al., 2015; Bryois et al., 2017; Balliu et al., 2019), proteomic (Lehallier et al., 2019), and metabolomic levels (Bunning et al., 2020; Hou et al., 2020). However, given the complex involvement of genetic, environmental, and lifestyle factors, the molecular changes that occur with aging remain poorly understood (López-Otín et al., 2013; Ahadi et al., 2020; Bunning et al., 2020).

In addition to molecular changes during aging, the host microbiome, consisting of diverse microbial communities, plays an important role in host physiology and metabolism (Sommer and Bäckhed, 2013; Manara et al., 2019). In particular, the gut microbiota is increasingly regarded as an ‘invisible organ’ in animals (Hill et al., 2017) and is associated with host physiology, immunity, neurological function, metabolism, and disease (Gilbert et al., 2018; Kurilshikov et al., 2021). Recent studies have also provided new insights into gut microbial trajectories associated with aging (Wilmanski et al., 2021; Zhang et al., 2021). For example, the gut microbiota develops rapidly from birth until 3 to 5 years of age (Hill et al., 2017; Roswall et al., 2021; Wilmanski et al., 2021), followed by a long period of relatively stability, then gradual changes with aging (O’Toole and Jeffery, 2015). Wilmanski et al. (2021) reported increasing compositional uniqueness of the gut microbiome as a component of healthy aging. Therefore, identifying age-related patterns in the gut microbiota may be useful for monitoring gut microbiome health and thus host health (Wilmanski et al., 2021).

Oral microbiota is also important because the oral cavity serves as the initial entry point for oral and gut microbial colonization (Xiao et al., 2020). In addition, oral bacteria are implicated in oral and dental health as well as many diseases that affect the elderly, such as non-oral cancers, cardiovascular disease, and Alzheimer’s disease (Hezel and Weitzberg, 2015; Olsen and Singhrao, 2015; Shoemark and Allen, 2015; Fan et al., 2018; Xiao et al., 2020). To date, however, most studies have focused on oral microbiome changes associated with oral disease (Chen et al., 2020; Nóvoa et al., 2020; Baker et al., 2021), with changes during aging still largely unexplored.

Research related to human aging presents several challenges, including ethical and practical difficulties in recruiting large cohorts for long-term research, long human lifespans, and differences in medical interventions, diet, and socioeconomic circumstances (Janiak et al., 2021). Non-human model organisms, such as roundworms, mice, and rats, can provide considerable data regarding the aging process (Kenyon, 2010; Campisi et al., 2019). However, given the distant evolutionary relationships between such species and humans, findings may not be directly transferable to human aging. In contrast, considering their close relationship to humans (Gibbs et al., 2007) and similar anatomy, physiology, and behavior (Lan et al., 2020; Yan et al., 2020), non-human primates, such as rhesus macaques (*Macaca mulatta*, RMs), are well-suited animal models and have been successfully used in aging-related studies (Mattison et al., 2017; Campisi et al., 2019; Zhou et al., 2020).

Studies have indicated that multi-omics analysis can effectively detect molecular changes during aging (Price et al., 2017; Schüssler-Fiorenza Rose et al., 2019; Zhou et al., 2019; Ahadi et al., 2020; Hou et al., 2020). Here, we used a multi-omics approach to profile the blood transcriptome and metabolome as well as the gut and oral microbiome in young adult (7–9 years old) and aged (>20 years old) Chinese rhesus macaques (*Macaca mulatta lasiota*, CRs). We examined the dynamics of molecular and microbiome changes and explored the changes in host–microbe interactions that occur during aging. This study should provide useful information to better understand and maintain the health of aged CRs and provide new insights into the mechanisms of aging.

## Materials and methods

### Sample collection

We collected anal swabs, oral swabs, and whole peripheral blood samples from young (*n* = 9, ages: 7–9 years) and old (*n* = 10, ages: 20–28 years) semi-captive CRs housed at Sichuan Green-House Biotech Co., Ltd. (Meishan, Sichuan, China) in a 2000-m^2^ open-air enclosure. All macaques were housed under the same conditions and fed the same diet.

PAXgene Blood RNA tubes were used to collect fresh blood samples. The samples were first stored at room temperature (18–20°C) for 4 h, then transferred to −20°C for 24 h, and finally preserved at −80°C until RNA extraction. Other blood samples were centrifuged on site to extract supernatants, which were then stored at −80°C for subsequent metabolome analysis and inflammatory factor measurement.

This study was approved by the Ethics Committee of the College of Life Sciences, Sichuan University, China (No. 20200327012 and No. 20210308001). All guidelines of the Management Committee of Experimental Animals of Sichuan Province, China (SYXK-Sichuan, 2019–192) regarding sample collection and use were strictly followed.

### RNA sequencing and differentially expressed gene analysis

Details on methods used are reported in our previous study (Lan et al., 2020). In brief, RNA samples were sent to Novogene (Beijing, China) for sequencing using the Illumina NovaSeq 6,000 platform with a paired-end sequencing length of 150 bp. The NGS QC Toolkit v2.3.3 (Patel and Jain, 2012) and HISAT2 v2.1.0 (Kim et al., 2015) were used for quality control and read mapping, and StringTie v1.3.6 (Pertea et al., 2015) was used to assemble transcriptomes and obtain raw read counts for each gene and transcript. Based on expression values, we used the DESeq2 R package (Love et al., 2014) to perform differential expression analysis. The DEGs were selected based on adjusted *p* ≤ 0.05 and log2 fold-change ≥1, resulting in 124 DEGs in the two age categories. Gene Ontology (GO) and Kyoto Encyclopedia of Genes and Genomes (KEGG) enrichment analyses were performed using g: Profiler (Raudvere et al., 2019) to functionally classify DEGs, with a Benjamini-Hochberg false discovery rate (FDR) ≤ 0.05 considered significant.

### Untargeted blood metabolomics analysis

The collected blood supernatants were sent to MetWare (Chengdu, China) for targeted metabolite analysis. After thawing the blood sample on ice, 300 μl of pure methanol was added to 50 μl of sample to remove blood protein. The samples were then centrifuged at 12000 *g* and 4°C several times (centrifuged 10 min → collected the supernatant → centrifuged 5 min → stored at 20° for 30 min → centrifuged 3 min). In total, 150 μl of supernatant was analyzed using high-performance liquid chromatography-electrospray tandem mass spectrometry (HPLC-ESI-MS/MS; UPLC, ExionLC AD; MS, QTRAP 6500 + System, Sciex). All chemicals used, including methanol (Merck), acetonitrile (Merck), formic acid (Aladdin), and standards (BioBioPha/Sigma-Aldrich), were chromatographically pure. Analyst v1.6.3 was used to analyze mass spectrometry data, in a qualitative analysis of the metabolites according to the retention time of the detected substance and secondary spectral data based on the MetWare database.

Each metabolite was subjected to a two-tailed unpaired t-test assuming unequal variances and using the q-value package (for total metabolites) to correct for false discovery.

### Metagenomic analysis

Total DNA from the swabs was extracted using a Tiangen DNA Stool Mini Kit (Tiangen Biotech Co., Ltd., China) and sent to Novogene (Beijing, China) for sequencing using the Illumina NovaSeq 6,000 platform with a paired-end sequencing length of 150 bp. After sequencing, adapters and low-quality raw reads were removed using Trimmomatic based on a four-base wide sliding window, with average quality per base >20 and minimum length 90 bp (Bolger et al., 2014). The CR potential sequences were removed using Bowtie2 (Langmead and Salzberg, 2012) as part of the KneadData pipeline^1^ with the RM reference genome (assembly Mmul_10). *De novo* assembly of the metagenomes from the quality-filtered Illumina reads was performed using MEGAHIT (Li et al., 2015) with the option “-t 96 –m 0.95 --min-contig-len 300.” Separate assembly of each sample was applied, rather than co-assembly of all samples, the advantages and disadvantages of which have been discussed previously (Pasolli et al., 2019). Gene prediction was performed using Prodigal (Hyatt et al., 2010) with the option “-p meta –g 11.” Non-redundant gene sets with thresholds of 95% similarity and 90% coverage of query sequences were constructed with CD-HIT (Fu et al., 2012) with the option “-c 0.95-aS 0.90.” The non-redundant genes were further translated into amino acid sequences. The amino acid sequences were aligned using DIAMOND (Buchfink et al., 2015) with the option “--id 80% --query-cover 70% --evalue 1e-5” in the Carbohydrate-Active enZYmes (CAZy) database (Lombard et al., 2014). Quantification of the non-redundant genes in each metagenome was performed using Salmon (Patro, 2017) with the option “--meta.” Total abundance of each gene type was determined by total abundance of all genes mapped to the same gene type. Gene family and microbial metabolic pathway abundances were assessed using HUMANn3 (Franzosa, 2018) with the ChocoPhlAn and UniRef90 EC filtered databases (Suzek et al., 2007), and were normalized by copies per million (CPMs). Taxonomic labels of metagenomic sequences were assigned using Kraken2 (Wood and Salzberg, 2014) with the option “--use-mpa-style.” Taxon abundances were normalized by relative abundance. Differences in taxon, functional gene, and metabolic pathway abundances were determined using Linear discriminant analysis effect size (LEfSe) analysis (Segata et al., 2011). Antibiotic resistance genes (ARGs) were quantified using ShortBRED (Kaminski et al., 2015). In brief, the shortbred_identify.py script was used to produce a FASTA file of markers using the ARG sets in the Comprehensive Antibiotic Resistance Database (CARD; Alcock et al., 2019) as proteins of interest and UniRef90 sequences as reference proteins, and the shortbred_quantify.py script was used to quantify the abundance of ARGs in the metagenomes. The ARG and gut microbiome abundance network was drawn using Cytoscape (Otasek et al., 2019).

### Diversity analyses

The taxonomic abundance table generated using Kraken2 was used as input for QIIME2 (Bolyen et al., 2019). The QIIME2 diversity plugin was used to calculate alpha (α) diversity (within sample diversity; Bolyen et al., 2019). The QIIME2 plugin DEICODE (Martino et al., 2019) was used to calculate beta diversity with feature loadings.

### Association analysis between multi-omics

To assess possible relationships between microbial genera, DEGs, and differentially expressed metabolites (DEMs), Spearman correlation analysis was performed based on the combined dataset of all variables. As an example, to determine whether microbe M is related to host genes *X*, *Y*, and *Z*, respectively, we calculated the correlation between the abundance of M and expression levels of *X*, *Y*, and *Z* in the samples, thereby obtaining three correlation values for microbe M, i.e., M ~ X, M ~ Y, and M ~ Z. This was carried out for all microbes and DEGs (and metabolites), with the top 100 relationships showing the highest relevance selected for display. All *value of p*s were corrected using the python model statsmodels. Stats. multitest. Multipletests (Supplementary Table 2).

### Inflammatory factor measurements

To quantify the levels of interleukin 21 (IL-21) and interleukin 27 (IL-27) in the two groups, enzyme-linked immunosorbent assays (ELISA) were performed using an ELISA kit (JM-10921 M2 and JM-10916 M2, Jingmei, Jiangsu, China). Data were analyzed using Wilcoxon rank tests, with *p* < 0.05 considered significant.

## Results

### Gut microbiota

**Figure 1:**
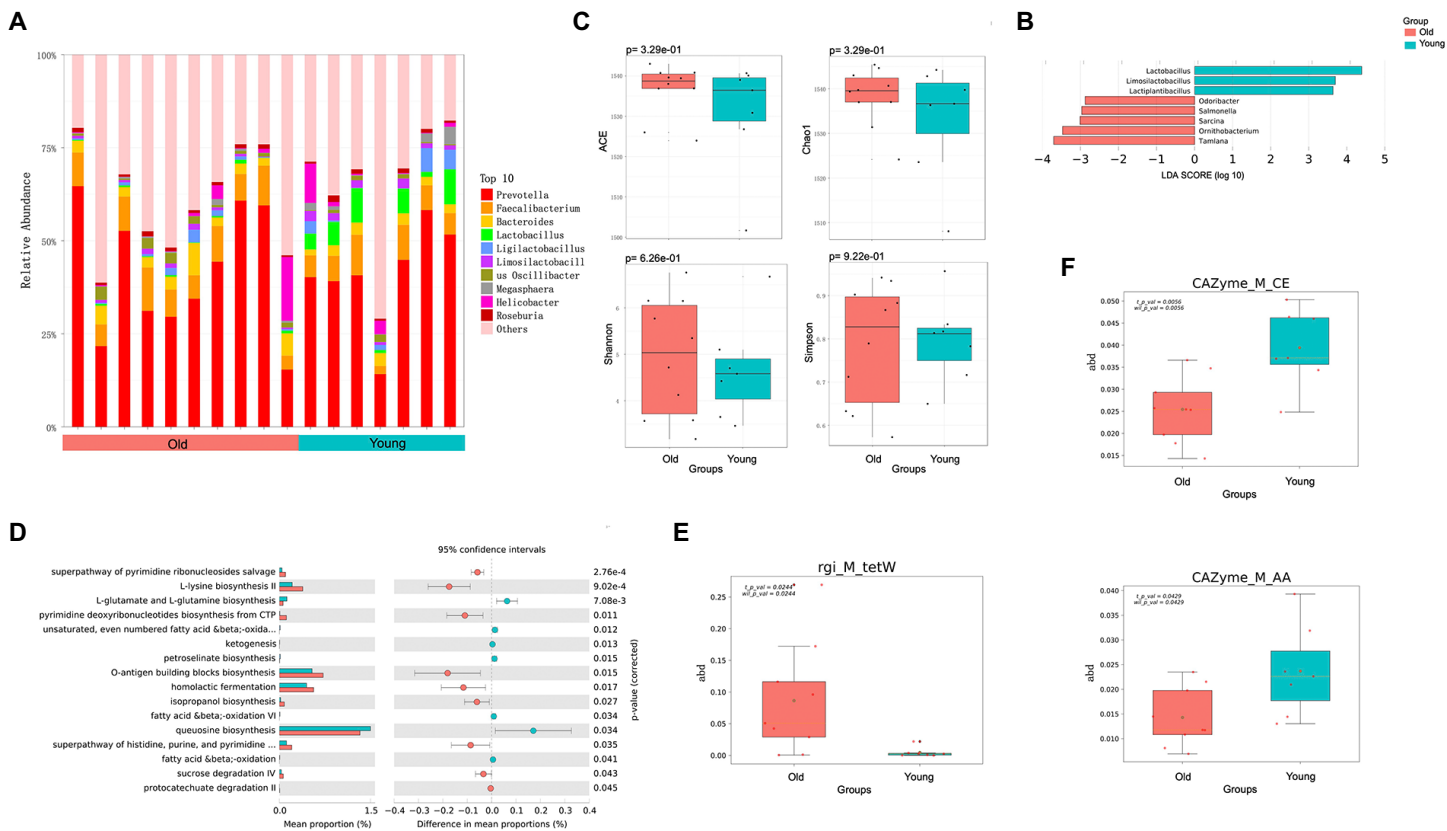
Metagenomics analysis of gut microbiota. **(A)** Top 10 most abundant genera in gut microbiome between young and old groups. **(B)** Differential analysis of gut microbial composition in young and old groups. **(C)** Alpha (α) diversity estimates between young and old groups. **(D)** Differential analysis of gut microbial function in young and old groups. **(E)** Differential analysis of gut microbial ARGs in young and old groups. **(F)** Differential analysis of gut microbial CAZy enzyme in young and old groups. *p <* 0.05 was considered significant.

To characterize differences in gut microbial composition, we compared relative abundance of gut bacteria between old and young CRs using the Wilcoxon rank sum test, which showed enrichment in different bacterial lineages. At the genus level, *Prevotella*, *Faecalibacterium*, and *Lactobacillus* were dominant in both groups (Figure 1A). Compared to the young group, the relative abundances of *Lactobacillus*, *Lactiplantibacillus* and *Limosilactobacillus* were lower in the old group (*p* < 0.05 and LDA > 2), while the relative abundances of *Salmonella*, *Odoribacter*, *Ornithobacterium*, *Tamlana*, and *Sarcina* were higher in the old group (*p* < 0.05 and LDA > 2; Figure 1B). Comparing α-diversity, results showed no significant differences in the four indices (ACE, Chao1, Shannon, and Simpson) between the two groups (*p* > 0.05; Figure 1C).

To characterize the global function of the gut microbiome and compare functional differences between young and old CRs, the abundances of microbial gene families and metabolic pathways were quantified using HUMANn3. Results indicated that the young CR gut microbiome was mainly enriched in aerobactin biosynthesis-related pathways such as L-lysine biosynthesis II, O-antigen building blocks biosynthesis, and pyrimidine deoxyribonucleotides biosynthesis, while the old CR gut microbiome was primarily enriched in L-glutamate and L-glutamine biosynthesis, ketogenesis, and petroselinate biosynthesis. Interestingly, the homolactic fermentation pathway was significantly enriched in the young CRs compared to the old CRs, while the opposite pattern was observed in the oral microbiome (Figure 1D).

Identification and quantification of ARGs showed that the tetracycline resistance *tet*W gene, was more abundant in the gut microbiome of old CRs than young CRs (*p* < 0.05; Figure 1E). Furthermore, carbohydrate esterases (CEs) and auxiliary activities (AAs) of the CAZy enzyme family were significantly down-regulated in aged CRs, indicating decreased carbohydrate metabolism in the older monkeys (*p* < 0.05; Figure 1F).

### Oral microbiota

**Figure 2:**
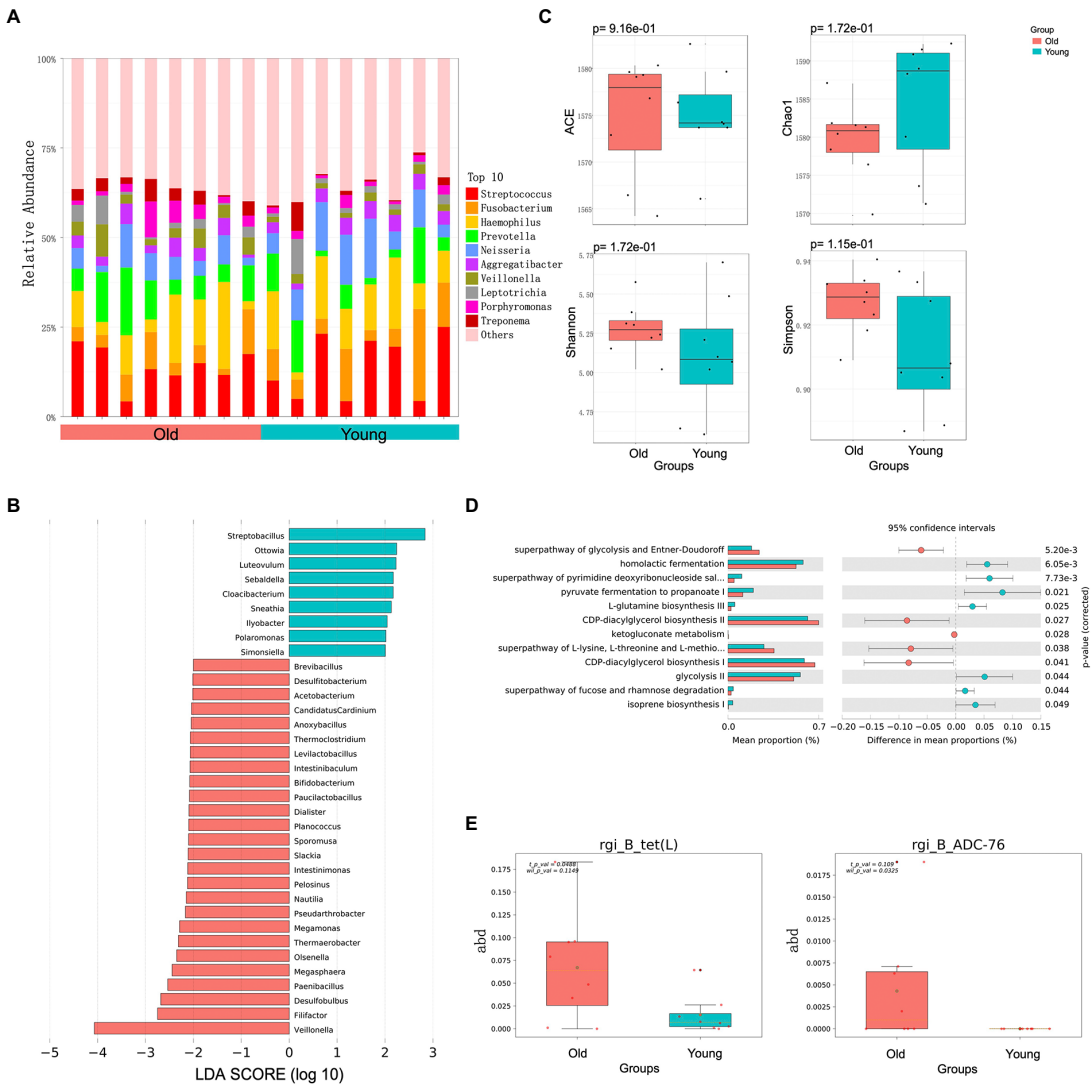
Metagenomics analysis of oral microbiota. **(A)** Top 10 most abundant genera in oral microbiome between young and old groups. **(B)** Differential analysis of oral microbial composition in young and old groups. **(C)** Alpha (α) diversity estimates between young and old groups. **(D)** Differential analysis of oral microbial function in young and old groups. **(E)** Differential analysis of oral microbial ARGs in young and old groups. *p* < 0.05 was considered significant.

We also identified and characterized differences in the oral microbiota of young and old CRs. Unlike the gut microbiota, however, the distribution of dominant microflora in the oral cavity of young and old CRs was similar, while the less abundant microflora showed considerable differences (Figure 2A). At the genus level, nine genera were significantly more abundant in the young CRs (*p <* 0.05 and LDA > 2), including *Ottowia*, *Simonsiella*, *Streptobacillus*. In contrast, 26 genera were more abundant in the old CRs (*p* < 0.05 and LDA > 2), including *Veillonella*, *Filifactor*, *Paenibacillus*, *Olsenella*, *Dialister*, *Sporomusa*, and *Bifidobacterium* (Figure 2B). No significant differences were found in α-diversity, consistent with the gut microbiota results (Figure 2C).

In addition to microbial composition, oral microbiota function and ARGs showed significant differences between young and old CRs. The young CR oral microbiome was enriched in several metabolic-related pathways, including CDP-diacylglycerol biosynthesis I/II, superpathway of glycolysis and Entner-Doudoroff, and superpathway of L-lysine, L-threonine, and L-methionine biosynthesis I. In contrast, the old CR oral microbiome was primarily enriched in homolactic fermentation, pyruvate fermentation to propanoate I, and glycolysis-related pathways (Figure 2D). For oral microbiota ARGs, the *tet* (L) gene, encoding resistance to tetracycline antibiotic, and the *ADC-76* gene, encoding resistance to cephalosporin, were more abundant in old CRs than in young CRs (*p* < 0.05; Figure 2E). However, there were no significant differences in the six CAZy enzyme family modules between the two groups (*p* > 0.05).

### Host metabolome

After removing unqualified samples, whole peripheral blood samples from 18 CRs (10 old and eight young CRs) were collected and secondary metabolite species were determined by HPLC-ESI-MS/MS. All metabolites were annotated using KEGG. Based on the local metabolite database, qualitative and quantitative mass spectrometry analyses were conducted on the sample metabolites. In total, 644 metabolites were identified, including 14 sugars, 56 amino acids and their derivatives, and 45 organic acids and their derivatives. All raw data for the detected metabolites are shown in Supplementary Table 1.

**Figure 3:**
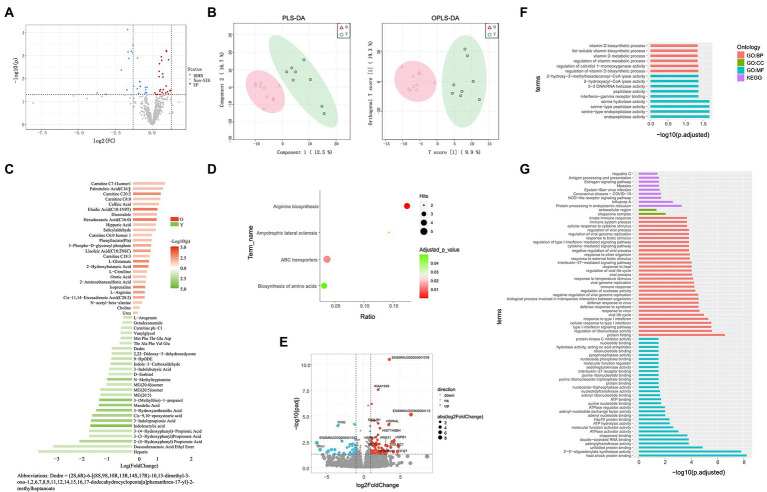
Blood metabolome and blood transcriptome analyses. **(A)** Volcano plots of metabolomes between young and old groups. **(B)** Partial least squares discriminant analysis (PLS-DA) and orthogonal partial least squares discriminant analysis (OPLS-DA) score plots based on metabolic profiles. **(C)** Differential abundance of blood metabolites in young and old groups (VIP ≥ 1, *p* < 0.05). **(D)** Enrichment analysis of differentially abundant pathways in young and old groups (*p* < 0.05). **(E)** Volcano plots of DEGs in young and old groups (log fold-change ≥1, *p* < 0.05). **(F)** GO and KEGG pathway enrichment analyses of up-regulated DEGs in old group (*p* < 0.05). **(G)** GO and KEGG pathway enrichment analyses of down-regulated DEGs of old group (*p* < 0.05).

Based on certain criteria (*p* < 0.05 and variable importance in projection (VIP) > 1), 53 significant DEMs were identified between the old and young groups (26 up-regulated and 27 down-regulated in old CRs; Figures 3A,B). Both CARs and FFAs were the major up-regulated DEMs in old CRs and included carnitine C18: 3, carnitine C20: 2, hexadecanoic acid (C16: 0), palmitoleic acid (C16: 1), and cis-11,14-eicosadienoic acid (C20: 2), which were enriched in the arginine biosynthesis, ABC transporter, amyotrophic lateral sclerosis, and biosynthesis of amino acid pathways. Indole and its derivatives, organic acid and its derivatives, and phenolic acids were the top three classes down-regulated in the old group and included 3-indolepropionic acid, indole-3-carboxaldehyde, indoleacrylic acid, and 3-hydroxyanthranilic acid. However, the down-regulated DEMs were not enriched in any pathway (Figures 3C,D).

### Host transcriptome

Whole peripheral blood transcriptomes of 15 CRs (nine old and six young CRs) were sequenced. The 524.5 million clean reads obtained after removing adaptor sequences and low-quality reads were aligned to the rhesus macaque reference genome (MMUL_10) separately with an average mapping rate of 95.55% per sample. All samples were processed using the same bioinformatics pipeline. After removing low-expression genes, the reads were assembled into 19,902 known genes.

In total, 124 genes were identified as DEGs between the groups (Figure 3E), including 44 up-regulated DEGs and 80 down-regulated DEGs in the old group compared to the young group. To clarify the biological roles of the DEGs, we performed GO and KEGG pathway enrichment analyses of the up-regulated and down-regulated DEGs separately. For GO enrichment analysis, the old down-regulated DEGs were mainly enriched in protein binding-or nucleotide binding-related molecular function (MF) categories, such as heat shock protein binding (GO: 0031072) and adenyl nucleotide binding (GO: 0030554). In the biological process categories, the old down-regulated DEGs were mainly enriched in immune response and interferon signaling pathways, such as interleukin-27-mediated signaling pathway (GO: 0070106), defense response to bacterium (GO: 0042742), Toll-like receptor 3 signaling pathway (GO: 0034138), and type I interferon signaling pathway (GO: 0060337). Based on KEGG enrichment analysis, the old CR down-regulated DEGs were significantly enriched in nine pathways, including NOD-like receptor signaling pathway (KEGG: 04621) and antigen processing and presentation (KEGG: 04612; Figure 3G). For analysis of old CR up-regulated DEGs, only GO terms related to vitamin D biosynthesis were enriched, including regulation of calcidiol 1-monooxygenase activity (GO: 0060558) and vitamin D biosynthetic process (GO: 0042368; Figure 3F).

### Association analysis between multi-omics

**Figure 4:**
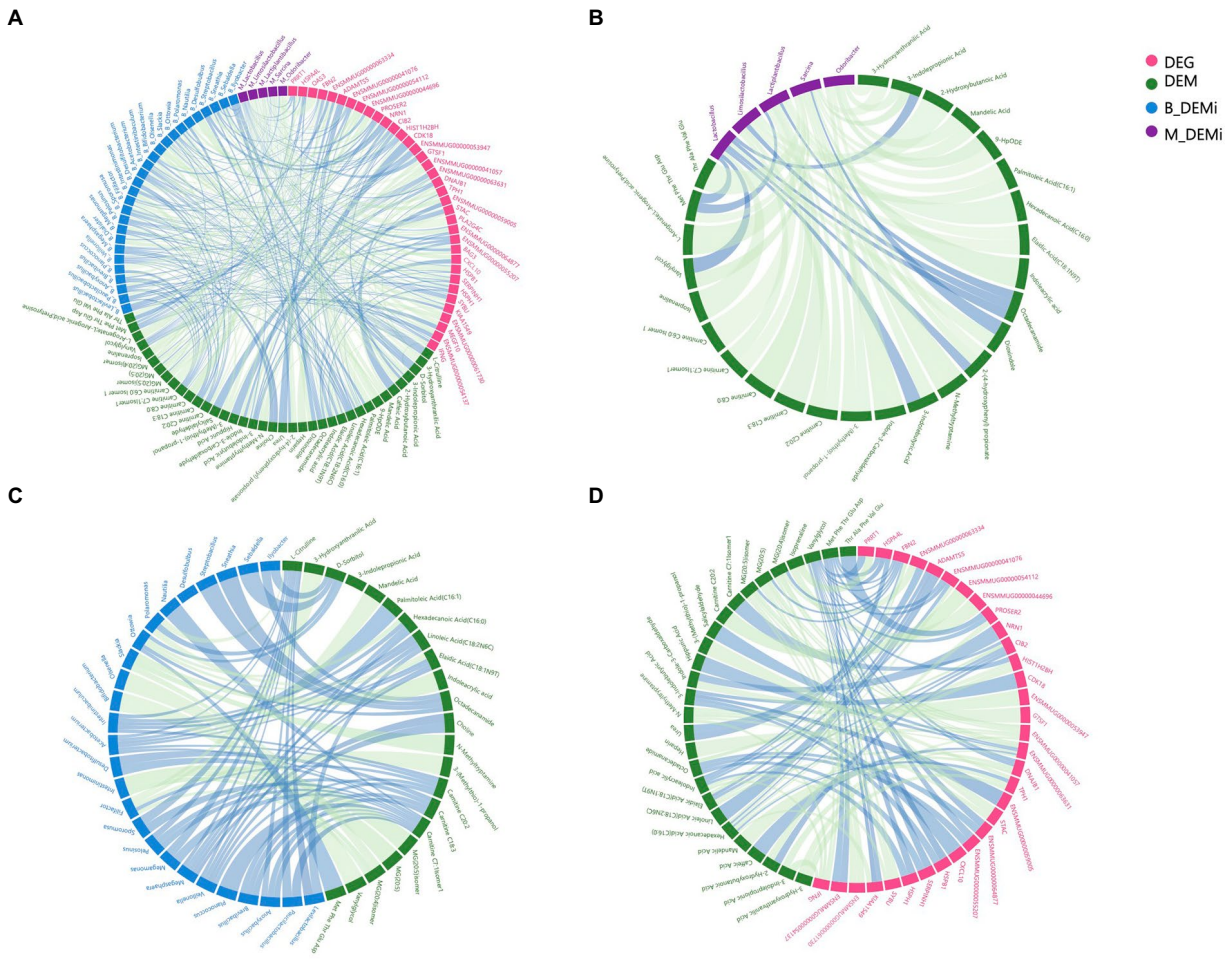
Association analysis among multi-omics. **(A)** Correlation network of variables that differed significantly between young and old groups of each omics. **(B)** Correlation network of differential gut microbial genera and DEMs. **(C)** Correlation network of differential oral microbial genera and DEMs. **(D)** Correlation network of DEGs and DEMs. DEGs: differentially expressed genes; DEMs: differentially expressed metabolites; B_DEMi: differentially expressed oral microbiota; M_DEMi: differentially expressed gut microbiota. All nodes and edges of correlation networks, with lines indicating significant correlations (*p* < 0.05). Blue lines indicate positive correlation and green lines indicate negative correlation.

To reveal host–microbe interactions, metabolites were used as the core factor to link differential microbial genera, DEMs, and DEGs. We performed Spearman correlation analysis, which generated a correlation network between the variables that differed significantly between the young and old CRs (Figure 4A). The correlation network between differential gut microbial genera and DEMs contained 36 nodes and 53 edges, and each of the top gut microbial genera had associations with more than four differential metabolites were *Sarcina*, *Lactiplantibacillus*, *Limosilactobacillus*, *Odoribacter*, and *Lactobacillus* (Figure 4B). As shown in Figure 4C, the correlation network between differential oral microbial genera and DEMs contained 51 nodes and 72 edges, and each of the top oral microbial genera associated with more than four differential metabolites were *Acetobacterium*, *Filifactor*, *Intestinibaculum*, *Megasphaera*, *Ottowia*, and *Veillonella*. The correlation network between DEGs and DEMs contained 64 nodes and 99 edges. We found that six DEGs, which each of them had more than four associations with differential metabolites, including *FBN2*, *ENSMMUG00000063631*, *ENSMMUG00000041057*, *IFNG*, *TPH1*, and *CIB2* (Figure 4D).

Analyses related to tryptophan metabolism appeared in each omics data. Thus, we next focused on the correlations between tryptophan metabolites (3-indolepropionic acid, indole-3-carboxaldehyde, indoleacrylic acid, and 3-indolebutyric acid), differential microbial genera, and DEGs. The DEGs showing the strongest association with differential tryptophan metabolites were *IFNG* and *ENSMMUG00000059005*. The differential gut microbial genera showing the strongest associations with differential tryptophan metabolites were *Sarcina* and *Lactobacillus*, and the differential oral microbial genus showing the strongest association with differential tryptophan metabolites was *Slackia* (Table 1).

**Table 1:** The correlation top three of tryptophan metabolites with DEGs and DEMi.

	source_id	target_id	Correlation coefficient	value of p
DEGs	*IFNG*	Indoleacrylic acid	−0.7889	0.000472
	*IFNG*	3-Indolepropionic Acid	−0.7853	0.000522
	*ENSMMUG00000059005*	3-Indolebutyric Acid	0.8786	1.63E-05
M_DEMi	M_*Sarcina*	3-Indolepropionic Acid	−0.7941	0.000239
	M_*Sarcina*	Indoleacrylic acid	−0.7794	0.000372
	M_*Lactobacillus*	3-Indolepropionic Acid	0.7029	0.002387
B_DEMi	B_*Slackia*	3-Indolepropionic Acid	−0.7643	0.000907
	B_*Slackia*	Indoleacrylic acid	−0.8	0.000342

### Inflammatory factor measurements

We determined the levels of immune factors IL-21 and IL-27 using an ELISA kit. As shown in Supplementary Figure S1, the level of IL-21 was significantly higher in the old group (*p* < 0.05), consistent with the transcriptome results, while the level of IL-27 showed no significant differences between the two groups (*p* > 0.05).

## Discussion

As CRs exhibit similar molecular and phenotypic changes to humans during aging, they are considered an excellent model species for studying cardiovascular (Zhou et al., 2020) and microbiota-associated human diseases (Chen et al., 2018; Janiak et al., 2021). Currently, our understanding of how the oral and gut microbiomes change in aged CRs and how these changes affect host gene expression and physiology during aging remains limited. Most previous studies on molecular changes during aging in non-human primates have been based on single omics data, e.g., transcriptome (Li et al., 2019; Yan et al., 2020; Zhou et al., 2020) and 16S rRNA gene amplicon profiling (Chen et al., 2018; Ebersole et al., 2020; Janiak et al., 2021). In the present study, we evaluated host–microbe interactions in aged CRs using a multi-omics approach.

first identified changes in oral and gut microbiome composition and function. The gut microbiota in young and old CRs was dominated by *Prevotella*, *Faecalibacterium*, and *Lactobacillus*, consistent with previous reports (Chen et al., 2018; Janiak et al., 2021). At the genus level, *Prevotella* was the most abundant in both young and old CRs, as found in humans on non-westernized diets associated with carbohydrates and fiber (Wu et al., 2011; Tett et al., 2019). *Lactobacillus* showed high abundance in both young and old CRs but was significantly more abundant in the young group. Based on 16S V4 rRNA amplicon sequences, Janiak et al. (2021) also detected significant differences in the relative abundance of *Lactobacillus* between infant and non-infant CRs, but not between other post-infancy age groups, suggesting that *Lactobacillus* may play an important role in digesting milk. *Lactobacillus* species are beneficial bacteria in the gut of humans and other mammals (Jia et al., 2010; Rossi et al., 2016; Valeriano et al., 2017), identified as important probiotics for gut health (Spinler et al., 2008). *Lactobacillus* species can inhibit the overgrowth of pathogens in the gut by producing various antibiotic factors, such as organic acid, hydrogen peroxide, and bacteriocin (Reid and Burton, 2002; Spinler et al., 2008; Linninge et al., 2019). Therefore, the significantly higher abundance of probiotics in young CRs may contribute to better gut health maintenance. The homolactic fermentation pathway was also enriched in the gut of young CRs, which inhibits the growth of intestinal saprophytes and ensures a healthy gut (Zhang et al., 2019).

In contrast, we identified two highly abundant genera (*Salmonella* and *Sarcina*) in the gut of old CRs associated with intestinal issues. *Salmonella* species are common food-borne pathogens (Bula-Rudas et al., 2015; Rivera-Chávez and Bäumler, 2015). In addition, *Sarcina* species can cause abomasal bloat and death in livestock, especially sheep and goats, and are associated with certain human diseases, including emphysema gastritis and gastric ulcers (Lam-Himlin et al., 2011). Functional analysis indicated that the L-glutamine biosynthesis III (PWY-6549) and L-glutamate and L-glutamine biosynthesis (PWY-5505) pathways were enriched in old CRs. These two pathways produce L-glutamine, which can induce cancer cells to undergo rapid growth (Daye and Wellen, 2012). Taken together, the significant decrease in probiotics and significant increase in pathogenic bacteria in old CRs may induce a variety of intestinal and other diseases.

Compared with the gut microbiome, the oral microbiome of CRs shows marked similarities to that of humans (Janiak et al., 2021). Similarly, we found that most of the top 15 genera in the oral microbiome of CRs were also core genera in the oral microbiome of healthy humans, e.g., *Streptococcus*, *Fusobacterium*, *Haemophilus*, *Porphyromonas*, and *Gemella* (Aas et al., 2008; Zaura et al., 2009; Krishnan et al., 2017; Chen et al., 2018). We also found a higher abundance of oral pathogens in old CRs, such as *Bifidobacterium*. *Bifidobacterium* is normally considered a probiotic, producing a variety of organic acids, including lactic acid, and showing beneficial effects, such as reducing the number of harmful bacteria in the gut (Gill et al., 2001; Manome et al., 2019). However, oral *Bifidobacterium* is recognized as a novel caries-associated bacterium, especially in children (Aas et al., 2008; Tanner et al., 2011; Kaur et al., 2013). In humans, frequently detected *Bifidobacterium* species in the oral microbiome include *B*. *dentium* (Munson et al., 2004) and *B. longum* (Nyvad and Kilian, 1990; Aas et al., 2008; Mantzourani et al., 2009). We detected 23 *Bifidobacterium* species in the oral microbiome of CRs, with *B. adolescentis* and *B. longum* found in higher abundance in old CRs than in young CRs. This is consistent with our observation that almost all aged individuals sampled showed evidence of caries or other oral disease. Functional pathway analysis showed that the homolactic fermentation pathway, which is related to lactic acid formation, was enriched in the oral microbiome of the old group. Lactic acid in the oral cavity is not beneficial, with long-term presence associated with tooth damage and corrosion (Jijakli and Jensen, 2019). Functional analysis also indicated that the L-glutamine biosynthesis III (PWY-6549) and L-glutamate and L-glutamine biosynthesis (PWY-5505) pathways were enriched in the oral and gut microbiomes of old CRs. Thus, the microbial characteristics of both the oral and gut microbiomes changed with age, likely increasing health risks for the host (Ahadi et al., 2020; Wilmanski et al., 2021).

*Filifactor alocis*, *Selenomonas*, and *Veillonella*, which are associated with periodontal diseases (Aruni et al., 2011; Barzilai et al., 2012; Antezack et al., 2021), were more abundant in the old CRs. *Filifactor alocis* has been used as a diagnostic marker for periodontitis due to its unique ability to tolerate oxidative stress and generate a strong pro-inflammatory response (Aruni et al., 2011; Moffatt et al., 2011). *Filifactor alocis* is associated with gingivitis, gestational diabetes, and oral squamous cell carcinoma (Gogeneni et al., 2015; Yang et al., 2018), but has not been found in healthy individuals (Schulz et al., 2019). *Selenomonas* is also associated with gingivitis and may be a potential biomarker for the onset of periodontitis (Tanner et al., 1996, 1998; Socransky et al., 1998; Antezack et al., 2021). Oral *Veillonella* is closely related to dental pulp, periapical infection, and chronic periodontitis, and can cause caries in cooperation with *Streptococcus mutans* (Barzilai et al., 2012). Comparing the oral microbiomes of young and old CRs, we identified several bacteria related to human oral diseases that were more abundant in aged CRs. These results suggest that aged CRs may face similar dental issues as humans, and thus could be an excellent model for studying oral diseases in humans.

We next investigated metabolite and gene expression levels in the plasma metabolome and blood transcriptome. Metabolomics is a popular approach to study aging, as metabolic changes are central to the aging process (Barzilai et al., 2012; López-Otín et al., 2013; Hou et al., 2020; Wilmanski et al., 2021). Metabolomics also reflects both genetic and nongenetic factors, as metabolites are influenced by the host, microbial activity, and environmental exposure (Rinschen et al., 2019; Bunning et al., 2020). In recent years, metabolomics has been successfully applied in the study of human aging, identifying many age-related biomarkers and biological pathways (Tanner et al., 1998; López-Otín et al., 2013; Price et al., 2017; Hou et al., 2020; Antezack et al., 2021; Wilmanski et al., 2021). However, few studies have explored metabolomics in non-human primates. Here, plasma metabolomics revealed significant differences at the single metabolite level between young and old CRs. We found that indole and its derivatives (indole-3-carboxaldehyde, 3-indolepropionic acid, and indoleacrylic acid), produced from tryptophan metabolism (Natividad et al., 2018), were significantly decreased in old CRs. These microbial tryptophan metabolites act as AhR ligands to increase IL-22 secretion (Zelante et al., 2013; Lamas et al., 2016). AhR is widely distributed in different mammalian cells and produces different effects by binding to different ligands, thus playing a vital role in immune and inflammatory responses, with its deletion leading to an increase in pro-inflammatory cytokines (Alexeev et al., 2018). Previous research has shown that the gut microbiota of *Anopheline* mosquitoes promotes anti-parasitic responses by participating in tryptophan metabolism (Feng et al., 2022).

In addition to indole and its derivatives, a variety of FFAs were also found in the aged CRs, including cis-11, 14-cicosadienoic acid (C20: 2), linoleic acid (C18: 2N6C), and hexadecanoic acid (C16: 0). Compared with other saturated FFAs, hexadecanoic acid (palmitic acid) is found at higher concentrations in human plasma (Richieri and Kleinfeld, 1995). High concentrations of palmitic acid can enhance serine 307 phosphorylation of insulin receptor substrate 1 (IRS1) through multiple mechanisms, leading to insulin resistance (Wei et al., 2018). High levels of palmitic acid can also induce excessive reactive oxygen species (ROS) production, resulting in mitochondrial and endoplasmic reticulum stress and fatty acid metabolism disorders (Tumova et al., 2016). Therefore, fatty acid metabolism disorders may occur in aged CRs.

Dysregulation of carnitine homeostasis is a prominent feature of fatty acid metabolism disorders (Bene et al., 2018). We detected higher abundance of carnitine in the old CR group. One of the main functions of carnitine is to transport long-chain fatty acids from the cytoplasm across the mitochondrial membrane into the mitochondrial matrix for β-oxidation, thereby generating cellular energy (Bene et al., 2018). Dysfunction in fatty acid metabolism can lead to a substantial accumulation of free carnitine and CARs (Möder et al., 2003; Zhang et al., 2014). Indeed, we found that the most significant changes in plasma metabolites in the aged CRs were the increases in free carnitine and CARs, including carnitine C18: 3, carnitine C6: 0 isomer 1, carnitine C8: 0, carnitine C20: 2, and carnitine C7: 1 isomer 1, further suggesting that older CRs may experience disturbances in fatty acid metabolism. This finding is similar to that reported in human studies showing that lipid metabolism disturbance can increase abnormal acylcarnitine levels, leading to obesity or diabetes (Mihalik et al., 2010).

The top three DEMs showing higher abundance in the aged CRs were all related to cardiovascular function and blood coagulation. Heparin is a commonly used anticoagulant for the prevention and treatment of venous thrombosis (Gray et al., 2008). 2-(4-hydroxyphenyl) propionic acid (2-HPPA CPD) can act on cyclooxygenase (COX) to regulate platelet aggregation (Yamanishi et al., 2008). Elevated levels of docosahexaenoic acid ethyl ester (DHA-EE) in the body can increase DHA levels, which play an important role in neurological and cardiac function (Martínez et al., 2000; Valenzuela et al., 2005), and thus DHA-EE intake may delay damage caused by aging. These results are consistent with our previous study, which showed that aged CRs have shorter coagulation times and higher coagulation factor II (FII) and VIII (FVIII) activity compared to young CRs (Zhou et al., 2020). The above results indicate that aged CRs may face the same problems as older humans, and thus are an appropriate model species for studying human cardiovascular disease (Zhou et al., 2020).

Consistent with the metagenome and metabolome results, transcriptome analysis detected several DEGs (*TPH1*, *IFNG*, *IL-21*, and *IL-27*) and signaling pathways related to inflammation and tryptophan metabolism. Tryptophan hydroxylase 1 (TPH1) is mainly expressed in enterochromaffin and other non-neuronal cells (Walther et al., 2003) and is the rate-limiting enzyme encoded by *TPH1* for tryptophan metabolism into serotonin (5-HT; Billing et al., 2019). 5-HT is a key neurotransmitter in the central nervous system and plays a role in controlling mood, sleep, and anxiety, and regulating gastrointestinal motility (Martin et al., 2017; Keating and Spencer, 2019). High levels of 5-HT and *TPH1* are observed in certain metabolic diseases (e.g., NAFLD and type 2 diabetes; Crane et al., 2015). Interferon-γ (IFN-γ), which is encoded by *IFNG*, is involved in tryptophan metabolism. Notably, IFN-γ can induce the synthesis of indoleamine-2, 3-dioxygenase, a tryptophan-catabolizing enzyme, converting tryptophan into kynurenine and reducing the level of tryptophan in plasma (Munn et al., 2005). IFN-γ is produced by natural killer (NK) and T cells and is considered a pro-inflammatory factor due to its strong macrophage activation potential (Seder et al., 1993). IL-21, which is encoded by *IL-21*, is secreted by follicular helper T cells (Tfh), peripheral helper T cells (Tph), and helper T cells (Th17). Stimulating T cell receptor (TCR) signals induces IL-21 production and IFN-γ release (Apetoh et al., 2010). In addition, IL-21 can promote the differentiation of CD4+ T cells into Th17 cells, which are robust producers of IL-21 (Manel et al., 2008). Dendritic cells (DCs) can express Toll-like receptors (TLRs), which can recognize cell wall components such as lipopolysaccharide, peptidoglycan, and lipoprotein. Therefore, TLRs are regarded as a bridge for DC activation caused by microbial components (Kawai and Akira, 2011). DCs also produce IL-27 in response to TLR activation (Molle et al., 2010). IL-27, which is encoded by *IL-27*, was originally thought to be a pro-inflammatory cytokine due to its structural homology with IL-12 and its ability to trigger IFN-γ production. However, Kastelein et al. (2007) found that IL-27 inhibits Th1, Th2, and Th17 cell responses and limits central nervous system inflammation. IL-27 also promotes the differentiation of type 1 regulatory T cells (Tr1 cells), which produce IL-10 to inhibit inflammation (Stumhofer et al., 2007). Our results showed that the expression levels of IL-21 and IFN-γ increased, whereas the expression level of IL-27 decreased in aged CRs. Furthermore, the Toll-like receptor TLR1: TLR2 and Toll-like receptor 3 signaling pathways, which recognize viruses and bacteria, were down-regulated in the aged CRs. In addition, the vitamin D biosynthetic process pathway, which directly and indirectly inhibits antigen processing and presentation and Th1 cell activation (Bartels et al., 2010), was up-regulated in aged CRs. These changes suggest that aged CRs may be more prone to tryptophan metabolism disorder and inflammation than young CRs.

Using multi-omics analysis, we observed many potential links among the detected differential microbiota, metabolites, and genes. We found that changes in metabolite composition in aged CRs were primarily caused by decreased abundance of beneficial genera (*Lactobacillus*, *Lactiplantibacillus*, and *Limosilactobacillus*) and increased abundance of pathogenic genera. Changes in metabolite composition may also have contributed to the increase in oral pathogenic genera (*Filifactor* and *Veillonella*). Among the DEGs associated with DEMs, *IFNG* and *TPH1* were both related to tryptophan metabolism. Correlation analysis showed that *IFNG* was strongly associated with tryptophan metabolites, consistent with studies showing that IFN-γ can accelerate tryptophan metabolism (Munn et al., 2005). At the same time, *Lactobacillus* in the gut was strongly associated with tryptophan metabolites, consistent with studies showing that *Lactobacillus* can metabolize tryptophan to produce indole-3-carboxaldehyde (Natividad et al., 2018). Zhang et al. (2022a,b) also found that *Lactobacillus* in the honeybee gut modulates host learning and memory behaviors *via* tryptophan metabolism regulation.

**Figure 5:**
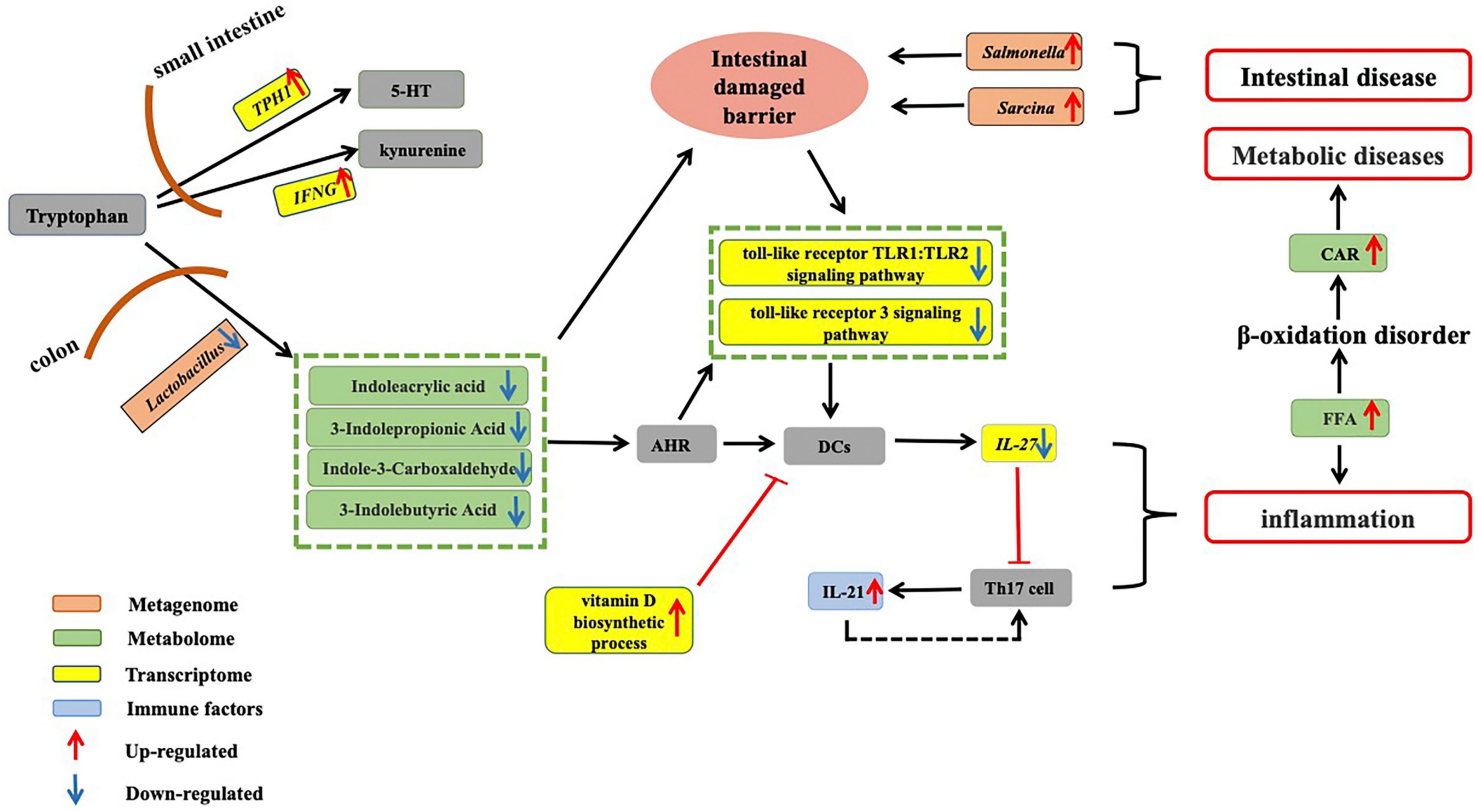
Dysregulation of tryptophan metabolism in aged CRs. Tryptophan obtained from food is absorbed in the small intestine. Up-regulation of key gene *TPH1*, which is involved in 5-HT synthesis, and up-regulation of IFN-γ, which is involved in the conversion of tryptophan into kynurenine, suggest a decrease in circulating tryptophan in aged CRs. On the other hand, in the colon, tryptophan is metabolized by gut microbiota to produce indole-3-carboxaldehyde, 3-indolepropionic acid, and indoleacrylic acid. Our results showed that *Lactobacillus* abundance decreased in old CRs, leading to a decrease in AhR ligands in the plasma. Down-regulation of the Toll-like receptor TLR1:TLR2 and Toll-like receptor 3 signaling pathways and up-regulation of vitamin D biosynthetic process pathway suggested that activity of DCs decreased, which may lead to a decrease in IL-27 expression and an increase in IL-21 expression. The decrease in microbial tryptophan metabolites may affect intestinal mucosal barrier integrity, allowing gut pathogens (such as *Salmonella* and *Sarcina*) to invade the host. Thus, these changes can induce the occurrence of inflammation and various gut diseases. Increased FFAs can lead to inflammation and accumulation of CARs, which may induce various metabolic diseases in aged CRs. CRs: Chinese rhesus macaques; AhR: Aryl hydrocarbon receptor; DCs: Dendritic cells; FFA: Free fatty acids; CAR: Acyl carnitine.

Based on our results, tryptophan metabolism appears to be critical for the physiological health of aged CRs (Figure 5). Most tryptophan obtained from food is absorbed in the small intestine and plays an important role in immune regulation and neuronal activity (Martin et al., 2017; Keating and Spencer, 2019). We found the key gene *TPH1* involved in 5-HT synthesis and IFN-γ involved in tryptophan convert into kynurenine were up-regulated, suggesting the increase in 5-HT and the decrease in circulating tryptophan in old CRs. In our study, the down-regulation of *SLC7A8*, which is involved in tryptophan transport, further confirmed the decrease in circulating tryptophan (Tina et al., 2019). Studies in healthy older people have shown that low circulating tryptophan is associated with several diseases, including neurodegenerative diseases, olfactory dysfunction, and immunological disorders (Denz et al., 1993; Iwagaki et al., 1995; Widner et al., 1999, 2000; Adachi et al., 2017).

We found that the abundance of *Lactobacillu*s, which is involved in tryptophan metabolism, decreased in aged CRs, leading to a decrease in AhR ligands (indoleacrylic acid, 3-indolepropionic acid, and indole-3-carboxaldehyde) in the plasma and down-regulation of the Toll-like receptor TLR1: TLR2 and Toll-like receptor 3 signaling pathways. Furthermore, IL-21 levels in the blood of aged CRs showed an increase. Based on these changes, we hypothesized that the decrease in *Lactobacillu*s may eventually lead to an increase in IL-21 by affecting AhR and DCs. In addition, up-regulation of the vitamin D biosynthetic process pathway may affect antigen processing and presentation. As a result, host defenses against bacteria and viruses are reduced, which may result in gut and oral pathogen invasion (such as *Salmonella*, *Sarcin*a, *Filifactor*, *Treponema*, *Selenomonas*, and *Veillonella*), leading to various gut and oral diseases.

Studies have shown that a lack of tryptophan can affect intestinal mucosal homeostasis, resulting in impaired intestinal immunity and altered gut microbial communities (Gao et al., 2018). Decreased tryptophan suggests that the intestinal mucosal barrier integrity was likely impaired in the aged CRs. Symptoms of intestinal barrier dysfunction and low-grade inflammation are common in metabolic diseases (Everard and Cani, 2013). Indeed, the increase in FFA and CAR accumulation indicated abnormal fatty acid metabolism, and the decrease in heparin, 2-HPPA CPD, and DHA-EE indicated cardiovascular disease in the aged CRs.

In conclusion, we applied transcriptomics, metagenomics, and metabolomics to study host–microbe interactions in aged CRs. Based on this multi-omics approach, we identified many potential links among differential microbiota, metabolites, and genes, indicating that aged CRs will likely experience multiple metabolic problems, immune disorders, and gut and oral diseases. Notably, we found that tryptophan metabolism is critical for the physiological health of aged CRs. Therefore, precise health management and care of aged CRs should be considered. Although the sample size was not large, our research showed that the multi-omics approach is robust and can reveal host–microbe interactions in non-human primates in the rapidly evolving fields of species conservation and molecular ecology. Thus, this approach could be applied to address species conservation and molecular ecology in other species.

## Data availability statement

The raw data of transcriptomes and metagenomes have been submitted to the China National GeneBank DataBase (CNGBdb) with the accession number CNP0002963. The identified metabolites were listed in the Supplementary Table.

## Ethics statement

This study was approved by the Ethics Committee of College of Life Sciences, Sichuan University (Nos. 20200327012 and 20210308001). We strictly obeyed the guidelines of the management committee of experimental animals of Sichuan Province, China (SYXK-Sichuan, 2019-192) in the sample collection and utility protocols.

## Author contributions

JX, YL, XW, and JW collected the samples. JX, YL, XW, XL, JW, and KS performed the bioinformatics analyses. JX and XW performed the experiments. JX, YL, XW, and ZF wrote the manuscript. JL, BY, and MS revised the manuscript. MS and ZF designed and supervised the study. All authors contributed to the article and approved the submitted version.

## Funding

This work was supported by the National Natural Science Foundation of China (Nos. 32070413 and 81900966).

## Conflict of interest

The authors declare that the research was conducted in the absence of any commercial or financial relationships that could be construed as a potential conflict of interest.

## Publisher’s note

All claims expressed in this article are solely those of the authors and do not necessarily represent those of their affiliated organizations, or those of the publisher, the editors and the reviewers. Any product that may be evaluated in this article, or claim that may be made by its manufacturer, is not guaranteed or endorsed by the publisher.

## Footnotes

^1^ http://github.com/biobakery/kneaddata
