# Supplementary material for: Multi-omics analysis reveals the host–microbe interactions in aged rhesus macaques
Source: Front Microbiol. 2022 Sep 27;13:993879. doi: 10.3389/fmicb.2022.993879 (PMC9551614; doi:10.3389/fmicb.2022.993879)
Supplement: Supplementary file 3 [file Table_3.DOCX]

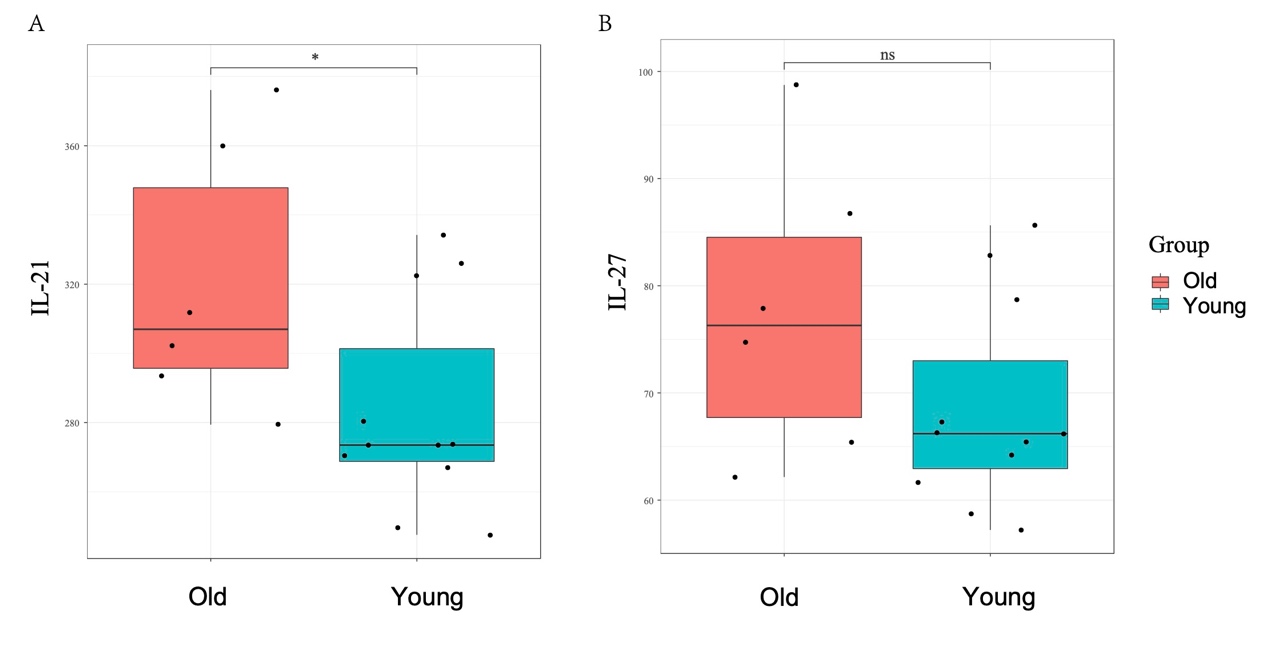


**Supplementary Figure S1. Inflammatory factor changes.** A) IL-21 level in two groups. B) IL-27 level in two groups. *P <* 0.05 was considered significant.
